# Analysis of the severity of occupational injuries in the mining industry using a Bayesian network

**DOI:** 10.4178/epih.e2019017

**Published:** 2019-05-11

**Authors:** Mostafa Mirzaei Aliabadi, Hamed Aghaei, Omid kalatpuor, Ali Reza Soltanian, Asghar Nikravesh

**Affiliations:** 1 Center of Excellence for Occupational Health (CEOH) and Occupational Health and Safety Research Center, Hamadan University of Medical Sciences, Hamadan, Iran; 2 Center of Excellence for Occupational Health (CEOH) and Research Center for Health Sciences, Hamadan University of Medical Sciences, Hamadan, Iran; 3 Department of Biostatistics and Epidemiology, School of Public Health and Modeling of Non-Communicable Diseases Research Center, Hamadan University of Medical Sciences, Hamadan, Iran; 4 Golgohar Mining and Industrial Company, Sirjan, Iran

**Keywords:** Occupational injuries, Accident, Bayesian approach, Mining industry

## Abstract

**OBJECTIVES:**

Occupational injuries are known to be the main adverse outcome of occupational accidents. The purpose of the current study was to identify control strategies to reduce the severity of occupational injuries in the mining industry using Bayesian network (BN) analysis.

**METHODS:**

The BN structure was created using a focus group technique. Data on 425 mining accidents was collected, and the required information was extracted. The expectation-maximization algorithm was used to estimate the conditional probability tables. Belief updating was used to determine which factors had the greatest effect on severity of accidents.

**RESULTS:**

Based on sensitivity analyses of the BN, training, type of accident, and activity type of workers were the most important factors influencing the severity of accidents. Of individual factors, workers’ experience had the strongest influence on the severity of accidents.

**CONCLUSIONS:**

Among the examined factors, safety training was the most important factor influencing the severity of accidents. Organizations may be able to reduce the severity of occupational injuries by holding safety training courses prepared based on the activity type of workers.

## INTRODUCTION

Despite considerable improvements in mining safety over recent years, accidents continue to occur, and mining is still one of the highest-risk industries worldwide [1,2]. Iran is a large, mineral-rich country, and according to national-level reports, occupational accidents have increased in mines in Iran [3]. Occupational accidents can have adverse impacts on workers, organizations, and communities [4,5].

Occupational injuries have long been regarded as one of the most negative outcomes caused by occupational accidents. The severity of occupational injuries is an important index for evaluating the outcome of accidents [6]. Various indices are used to assess the severity of occupational injuries, including the accident severity rate, the number of lost workdays, and the amount of damage to body parts [7,8]. The severity of occupational injuries is a multifactorial phenomenon, and several factors can contribute to it.

Studies have shown that both individual and organizational factors are related to the severity of occupational injuries [9-11]. Age, experience, and marital status are classified as individual factors, while factors such as job, activity type, and shift work are classified as organizational factors [5]. Examining the factors affecting injury severity in the workplace is helpful for minimizing the severity of accidents and reducing lost workdays.

In previous studies, researchers have used various type of regression approaches to analyze the severity of accidents. For instance, Mohammadfam et al. [5] applied structural equation modeling to analyze construction accident severity, and Onder [12] modeled the severity of occupational injuries using logistic regression. Despite the various advantages of regression approaches, it has some limitations, such as an inability to infer causal interactions, a restrictive model structure, and the ability to analyze only linear interactions [13]. To overcome those limitations of regression approaches, researchers have applied Bayesian network (BN) analysis as another powerful approach.

BN analysis is a powerful graphical approach for modeling causal relationships among variables. Each BN has a qualitative and a quantitative section [14]. The qualitative section consists of a set of nodes that represent the variables of study and directed arcs that indicate the causal relationships between variables. The quantitative section of a BN comprises conditional probability tables (CPTs) that determine the exact relationships among the variables analyzed in a study. Using belief updating, which is a unique characteristic of BN analyses, researchers can perform various types of inference, such as inter-causal, diagnostic, and predictive.

The BN approach has been used to analyze the severity of accidents in various fields, such as traffic accidents [15,16], however, the use of this approach to analyze occupational accident severity, especially in the mining sector, has been rare. Accordingly, the purpose of the present study was to analyze the severity of occupational injuries in mining accidents using the BN approach, with the goal of identifying strategies for improvement.

## MATERIALS AND METHODS

In this cross-sectional study, all reports of registered non-fatal occupational accidents that led to employee injuries within a 10-year period (2008-2017) were gathered from 10 large iron ore mines located in the south of Iran. As most previous studies have concentrated on fatal accidents in the mining sector, and less attention has been paid to non-fatal accidents, the present study investigated lost working days as an indicator of the severity of non-fatal accidents. Next, only occupational accident reports with complete data were considered. Finally, using a designed checklist, the required data was extracted.

### Variables

The variables of this study were selected based on the authors’ previous experiences and the results of the study by Soltanzadeh et al. [17]. In that study, the researchers used multiple linear regression to identify the associations of individual and organizational factors with the severity of accidents.

The accident reports and injured employees’ documents were reviewed and 11 variables were collected. One variable was considered as the response variable, and the remaining 10 variables as predictors. The number of workdays an injured worker took before returning to work (lost workdays) was selected as the response variable, because this variable indicates the severity of an injury due to an accident [18]. Age, marital status, experience, educational level, training, previous accident, work shift, day of the week, type of accident, and activity type of the injured employee were selected as predictor variables. To conduct a BN analysis, the states of each variable were defined (Table 1).

### Graphical structure of Bayesian network

After the variables and their states were defined, the graphical structure of a BN that represents the relationships among variables should be established. There are 2 ways to achieve this goal: first, to utilize expert knowledge; and second, to use a learning algorithm to create the graphical structure [19]. Because learning algorithms have disadvantages such as requiring a large data set, the applicability of this approach is limited, and expert knowledge may be preferable. Focus group analysis and the Dempster-Shafer theory are popular approaches for obtaining expert knowledge. In this study, we used the focus group approach, because the Dempster-Shafer approach involves a somewhat complicated procedure for obtaining answers from experts and performing the associated calculations, and the experts might not have been familiar with it.

### Conditional probability calculation

After the graphical structure of the BN was built, the next stage is to calculate the CPT for each node. A common approach for calculating CPTs is the parameter estimation process, which utilizes a data set to carry out the estimation [20]. Different algorithms have been utilized in the parameter estimation process. In this study, the expectation maximization (EM) algorithm [21,22] was utilized, since it is one of the most popular algorithms. The EM is an iterative approach for estimating the maximum likelihood of a set of parameters. This algorithm is popular for cases in which some random variables are hidden. In the present study, we utilized Netica version 5.24 (Norsys Software Corp., Vancouver, Canada) to perform the BN analysis.

The study protocol was approved by the Hamadan University of Medical Sciences (ethical code: IR.UMSHA.REC.1395.458).

## RESULTS

To establish the database in this study, a total of 425 accident cases were reviewed and analyzed. As described above, after selecting the response and predictor variables, we utilized knowledge of 4 safety experts (focus group) to determine the casual relationships among those variables. Based on this analysis, except for age, which had an indirect influence, the other variables were considered to have a direct effect on lost workdays. Furthermore, in addition to the direct effects of the experience and previous accident variables, they were considered to have an indirect effect on lost workdays, mediated by training.

**Figure 1.**
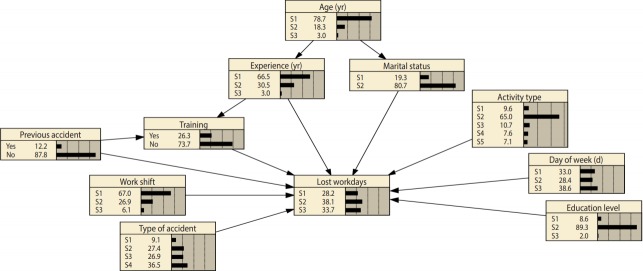
Network structure and prior probability states of variables (refer to Table 1).

After establishing the graphical structure of the BN, by using the EM algorithm and the established database, the CPTs of the variables were calculated. Figure 1 illustrates the network structure and the prior probability of the variables.

The prior probability of variables, as depicted in Figure 1, reflects the current status of the workplace with regard to the severity of accidents and other variables. As shown in this figure, 38.1% of injured workers had 1-5 lost workdays, 65.0% were technicians, 12.2% had experienced a previous accident, 73.7% had not taken a related safety training courses, 66.5% of them had less than 5 years of experience, 89.3% had a high school degree, more than 78.7% were younger than 30 years of age, and 80.7% of them were married. Additionally, 67.0% of accidents occurred during the morning shift and 38.6% of accidents occurred in the last 2 days of the week.

**Figure 2.**
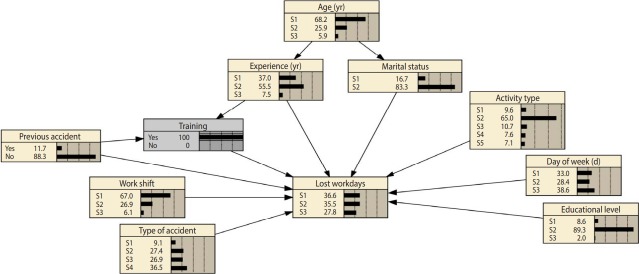
Setting evidence to the “training” node and the posterior probability states of other variables (refer to Table 1).

Belief updating, a unique characteristic of BN analysis, was utilized to assess the influence of changes in some variables on others. Applying this characteristic enables researchers to assess the sensitivity of one variable to changes in others, thereby determining the magnitude of the effects of variables for minimizing the consequences of accidents (with the goal of eliminating lost working days) and helping to select the best strategies for reducing the severity of injuries. This characteristic, which is based on the Bayes theorem, was fully described by Jensen & Nielsen [14]. An example of this characteristic is shown in Figure 2, in which the evidence is set to the “training” node. By comparing Figures 1 and 2, we can see changes in the probability of different states of other variables.

The sensitivity of lost workdays to other variables was assessed utilizing equation (1) and the method that was fully explained by Mohammadfam et al. [23].

(1) ∆p=plost workday = withoup lost workdaysvariable="a state"-plost workdays=without lost workdaysplost workdays="withour lost workdays"×100

Where, *p* (“lost workday”=without lost workdays|*variable*=“a state) is the probability of an accident without lost workdays given that other variables are in their various states (note: each state should be assessed separately) and *p* (*lost workdays*=“without lost workdays”) is the marginal probability of an accident without lost working days. For more information regarding this equation, please see Mohammadfam et al. [23].

The sensitivity analysis results are shown in Table 2. Having taken a training program related to workplace safety (44.76%) had the strongest influence on the severity of accidents. Workers’ experience (40.41%) and type of accidents (30.91%) also had considerable effects on the severity of the mining accidents. Workers’ marital status and the day of the week had the weakest effect on the severity of accidents.

## DISCUSSION

Occupational injuries are one of the worst negative outcomes of occupational accidents, especially mining accidents. Conducting quantitative causal analyses of the factors that affect the severity of accidents can help to reduce the severity of occupational accidents. The BN approach is a powerful graphical tool for quantitative analyses based on causal relationships among several variables [19,20,23]. The current study aimed to analyze occupational injuries involving mining accidents on the basis of individual and temporal factors through a BN, with the goal of finding strategies for improvement.

According to the results of the current study, 71.8% of occupational injuries led to at least one lost workday, which is comparable to the findings of a study conducted in the Turkish mining industry [12].

Among the variables included in the current study, training had the strongest influence on the likelihood of lost workdays. Furthermore, if workers were to take safety training courses, the percentage of accidents without lost workdays could increase to as high as 8.4%. Safety training programs play a crucial role in improving workers’ knowledge about the hazards of workplace [19]. Training has a positive effect on the reduction of injuries leading to lost time [24]. Ren et al. [25] found that safety education and training can increase workers’ job competencies, meaning that this factor has a crucial effect on avoiding severe outcomes of accidents.

However, it should be noted that only a well-designed safety training program can be effective in this regard [26]. Several studies have shown that it is possible for safety training not to have a significant effect on safety behavior and accidents due to deficiencies in design and implementation [27]. In this regard, Namian et al. [28] have emphasized the need for “training transfer elements,” which are necessary for the practical use of safety knowledge obtained from training programs in real daily situations. Top management commitment, supervisor support, feedback, incentives, and worker motivation are some such elements proposed by Namian et al. [28] for the construction industry. However, it should be noted that some of those elements are shared by both the construction and mining industries, while some others should be redefined for application in the mining industry.

Additionally, our sensitivity analysis indicated that certain variables related to individual characteristics, including workers’ experience and education level, had significant effects on the severity of accidents. The direct effects of individual factors on the severity of accidents have been well-documented [5]. Experienced workers are less likely to engage in unsafe behavior [19]. Our results are in line with those of a previous study [19] that found a higher rate of occupational accidents among low-experience workers. These findings imply that more experienced workers should be employed in areas with higher risk levels, because they are more familiar with those hazards and with ways of dealing with them to prevent associated accidents. Another inference is that managers should ensure that some experienced workers are present in each working group. Experienced workers should be distinguished from old workers, who are more prone to accidents such as slips, trips, and falls [29].

Other studies [10,11] have shown that variables such as workers’ age and experience can be crucial mediating factors in the severity of occupational accidents.

The sensitivity analysis showed that the type of accident had an important effect on the severity of accidents. A significant relationship has been shown between the outcome of an accident and the reason that it occurred (i.e., the type of accident) [30]. It has been reported that falling from height is one of the most common causes of accidents with severe outcomes [5,31].

Furthermore, the sensitivity analysis indicated that activity type had a significant effect on the severity of accidents. The activity type of workers is an important causal factor with a direct or indirect effect on the severity of occupational injuries [9,10,32]. Management can play a role in reducing both the occurrence and severity of accidents by preparing proper training programs and work procedures based on workers’ activity type [9,32,33]. In some activities, such as maintenance, where workers are exposed to high-risk conditions and high energy levels (e.g., working at height and maintenance of machines), the probability of severe accidents has been found to be higher [12]. Furthermore, providing safety training programs with due consideration of workers’ activity type and the types of hazards identified in the workplace can be considered as a way to minimize the severity of accidents. Moreover, experienced (not old) workers should be employed in these areas.

Finally, in the current study, a BN was utilized to find strategies for reducing the severity of occupational injuries caused by accidents at workplaces. In comparison with other approaches that are often utilized to investigate outcomes of accidents, such as structural equation modeling (SEM), the BN method has the benefit of enabling researchers to predict the intended outcome, whereas SEM is a powerful approach for explaining interrelationships between casual factors of accidents, and usually is not utilized for prediction.

Moreover, we suggest that future studies should utilize the BN approach to analyze the interactions and effects of other variables, such as unsafe acts, unsafe conditions, and additional organizational factors on accident outcomes.

Several strengths and limitations of the current study should be acknowledged. In the current study, numerous mining accidents were considered; hence, the sample size was a major strong point of the study. Another strong point was the use of a well-accepted graphical quantitative analytical approach (i.e., a BN). In this study we modeled the effects of some important variables on the severity of mining accidents; however, it would be very valuable if more individual and organizational factors, such as risk-taking behavior, job satisfaction, and inadequate supervision, were to be considered in future studies.

In conclusion, the results of the current study support the utility of applying the BN approach to scrutinize the severity of occupational injuries. The BN findings showed that safety training, activity type, and type of accidents were the most important factors influencing the severity of accidents. Therefore, by holding quality safety training with due consideration of workers’ activity type, organizations may be able to reduce the severity of occupational injuries in the mining sector.

## Figures and Tables

**Table 1.** States of study variables

Variable	States of variables
Age (yr)	S1: <30; S2: 30-40; S3: >40
Marital status	S1: single; S2: married
Experience (yr)	S1: <5; S2: 5-10; S3: >10
Educational level	S1: primary education; S2: high-school education; S3: academic education
Training	S1: “yes” for an injured employee who had taken a related safety training course; S2: “no” for an injured employee who had not taken a related safety training course
Previous accident	S1: “yes” for an injured employee who had experienced a previous accident; S2: “no” for an injured employee who had not experienced a previous accident
Work shift	S1: morning; S2: evening; S3: night
Day of week (d)	S1: first 2; S2: middle 2; S3: last 2
Type of accident	S1: electrical, fire, and explosion; S2: fall; S3: collision; S4: caught in/or between objects
Activity type	S1: machine operator; S2: technician; S3: simple worker; S4: driver; S5: service worker
Lost workdays	S1: no; S2: between 1 and 5; S3: more than 5

**Table 2.** The relative variation in the probability of accidents without lost workdays given various states^1^ of other variables

Variables	S1	S2	S3	S4	S5	Absolute mean of variation	Rank
Age	-17.3	30.6	21.2	-	-	23.04	6
Marital status	-2.1	5.8	-	-	-	3.94	10
Experience	21.7	41.2	58.3	-	-	40.41	2
Educational level	-20.1	16.9	35.6	-	-	24.21	5
Training	60.6	-28.9	-	-	-	44.76	1
Previous accident	23.9	-8.8	-	-	-	16.36	7
Work shift	-5.8	-9.3	19.9	-	-	11.69	8
Day of week	-3.4	4.1	10.3	-	-	5.92	9
Type of accident	-20.3	26.5	-35.2	41.6	-	30.91	3
Activity type	-21.0	22.9	26.5	-35.4	-33.7	27.91	4

Values are presented as %.

1 State of variable (refer to Table 1).
